# The Assessment of Antimicrobial Resistance in Gram-Negative and Gram-Positive Infective Endocarditis: A Multicentric Retrospective Analysis

**DOI:** 10.3390/medicina59030457

**Published:** 2023-02-24

**Authors:** Camelia Melania Budea, Marius Pricop, Ion Cristian Mot, Florin George Horhat, Kakarla Hemaswini, Raja Akshay, Rodica Anamaria Negrean, Andrada Licinia Oprisoni, Cosmin Citu, Bogdan Andrei Bumbu, Abduljabar Adi, Ibrahim Khan, Adelina Mavrea, Iulia Bogdan, Adrian Vasile Bota, Roxana Manuela Fericean, Iosif Marincu

**Affiliations:** 1 Department of Ear-Nose-Throat, “Victor Babes” University of Medicine and Pharmacy Timisoara, 300041 Timisoara, Romania; 2 Methodological and Infectious Diseases Research Center, Department of Infectious Diseases, “Victor Babes” University of Medicine and Pharmacy, 300041 Timisoara, Romania; 3 Discipline of Oral and Maxillo-Facial Surgery, Faculty of Dental Medicine, “Victor Babes” University of Medicine and Pharmacy Timisoara, 300041 Timisoara, Romania; 4 Multidisciplinary Research Center on Antimicrobial Resistance (MULTI-REZ), Microbiology Department, “Victor Babes” University of Medicine and Pharmacy, 300041 Timisoara, Romania; 5 Malla Reddy Institute of Medical Sciences, Hyderabad 500055, India; 6 Faculty of Medicine and Pharmacy, University of Oradea, 410073 Oradea, Romania; 7 Department of Pediatrics, Discipline of Pediatric Oncology and Hematology, “Victor Babes” University of Medicine and Pharmacy Timisoara, 300041 Timisoara, Romania; 8 Department of Obstetrics and Gynecology, “Victor Babes” University of Medicine and Pharmacy Timisoara, 300041 Timisoara, Romania; 9 Department of Dental Medicine, Faculty of Medicine and Pharmacy, University of Oradea, 410073 Oradea, Romania; 10 Faculty of General Medicine, Baskent University, 06790 Ankara, Turkey; 11 Department of Internal Medicine I, Cardiology Clinic, “Victor Babes” University of Medicine and Pharmacy Timisoara, 300041 Timisoara, Romania

**Keywords:** endocarditis, antibiotics, antimicrobial resistance, bacterial infections

## Abstract

*Background and Objectives:* Multidrug-resistant microorganisms have made treating bacterial infections challenging. Resistance to antibiotics is expected to overcome efforts to produce new, effective antibacterial medication that is lifesaving in many situations. Infective endocarditis (IE) is a life-threatening infection that affects 5–15 per 100,000 patients annually and requires rapid antibiotic therapy to prevent morbidity and mortality. *Materials and Methods:* The present research assessed IE cases over five years, from a multicentric database, with the main objective of determining the degree of antibiotic resistance in these patients, stratified by Gram-positive and Gram-negative bacteria. *Results:* Bad oral hygiene was present in 58.6% of patients from the Gram-negative group (vs. 38.7% in the Gram-positive group). Non-valvular heart disease was identified in approximately 40% of all patients, and valvopathies in approximately 20%. It was observed that 37.9% of Gram-negative IE bacteria were resistant to three or more antibiotics, whereas 20.7% were susceptible. Among Gram-positive infections, *S. aureus* was the most commonly involved pathogen, with a multidrug-resistant pattern in 11.2% of patients, while *Acinetobacter baumannii* had the highest resistance pattern of all Gram-negative pathogens, with 27.4% of all samples resistant to three or more antibiotics. Patients with Gram-negative IE were 4.2 times more likely to die. The mortality risk was 4 times higher when bacteria resistant to two or more antibiotics was involved and 5.7 times higher with resistance patterns to three or more antibiotics than the reference group with no antibiotic resistance. Peripheral catheters were the most common cause of multi-resistant IE, followed by heart surgery, dental procedures, and ENT interventions. *Conclusions:* Even though Gram-positive infections were the most frequent (83.0% of all cases), Gram-negative IE infections are substantially more deadly than Gram-positive IE infections. However, it was also observed that patients with Gram-negative infections were more likely to have underlying comorbidities, be institutionalized, and be underweight. Although the Gram-negative infections were more severe, their resistance patterns were similar to Gram-positive bacteria. As resistance patterns increase, more efforts should be made to prevent a healthcare catastrophe. At the same time, careful prophylaxis should be considered in patients at risk, including those with central catheters, undergoing dental procedures, and with poor oral hygiene.

## 1. Introduction

One of the most significant healthcare dangers of the modern era is antimicrobial resistance, which has become a worldwide public health emergency [1,2]. Certain bacterial strains have developed resistance to almost every antibiotic available. As a result, developing novel antibacterial agents is of absolute necessity. To direct and encourage the research and development of new antibiotics, a ranking list was created based on the pathogens’ classification, resistance features, and level of urgency, with critical, high, and medium priority categories [3].

The vast majority of Gram-negative bacteria are considered to be pathogens. Distinguished by their unique cell envelope structure, Gram-negative bacteria are associated with a higher mortality and morbidity rate on a global scale [4,5,6]. On the other side, many different Gram-positive bacteria are commonly known for some of the most dangerous community and healthcare-associated resistant infections [7]. From this genre, the Methicillin-resistant bacteria *Staphylococcus aureus* (MRSA), *Enterococcus faecium*, resistant to vancomycin, and *Streptococcus pneumonia*, resistant to several antibiotics, are of special concern [8,9].

Although the Gram classification of bacteria is based on a staining method, the particular characteristics of the involved microorganisms allowing them to be colorized are also involved in different pathogenic mechanisms and severities of infection. Endocarditis is considered a rare but severe infection of the heart valves that can occur on native or damaged valves, triggered by events like dental procedures, ear-nose-throat interventions, venous drug use, catheter insertion, and other interventions that lead to a release of pathogens into the bloodstream, allowing them to attach to the heart valves [10,11,12,13].

Infections with Gram-positive streptococci, staphylococci, and enterococci account for the great majority of IE cases, where *Staphylococcus aureus* alone accounts for around 30% of infections [14]. Other common oropharyngeal colonizers, such as the HACEK organisms (*Haemophilus* species, *Aggregatibacter* species, *Cardiobacterium hominis*, *Eikenella corrodens*, and *Kingella* species), most of them being of Gram-negative classification, account for approximately 20% of all endocarditis infections [15,16]. The underlying etiology of IE can be considered as community-acquired or nosocomial early prosthetic valve endocarditis within the first 60 days after surgery or following recent angiography, hemodialysis, or extra-cardiac surgical procedures [17]. *S. aureus* causes around 50% of all nosocomial IE, while the less pathogenic coagulase-negative staphylococci generally originate from intravascular devices or newly implanted prosthetic valves. Enterococci are involved in about 15% of the nosocomial cases of IE and 20% of the non-nosocomial cases [18,19].

Community-acquired infections are more likely to occur in the presence of immunosuppression, intravenous drug use, poor dentition, degenerative valve disease, and rheumatic heart disease [20,21]. Intravenous drug use, which accounts for almost 10% of infectious endocarditis cases, implies frequent inoculation with skin flora such as *S. aureus* and *S. epidermidis*, with *S. aureus* displaying a preference for healthy, native tricuspid valves [22]. About 20% of community-acquired infections are caused by the *Viridans* group streptococci [23]. Infections *with Streptococcus gallolyticus* organisms should traditionally prompt suspicions of colon cancer [24].

Although the epidemiology of infective endocarditis is well known, the causative bacteria can be prone to variability as in other types of infections, based on the population structure and medical conditions; the classification of the bacteria often influences this, the antimicrobial sensitivity status is the most influential aspect [25,26]. Therefore, the main objective of the current study was to observe the pattern of antimicrobial resistance among patients with IE, stratified by Gram-positive and Gram-negative bacteria. The study’s secondary objective was to describe the clinical characteristics of the patients with IE, considering the low incidence of the disease.

## 2. Materials and Methods

### 2.1. Study Design and Ethical Considerations

The current research was designed as a retrospective cohort study of hospitalized patients with a definitive diagnosis of infective bacterial endocarditis. Patients included in the study were admitted to different hospitals affiliated with the “Victor Babes” University of Medicine and Pharmacy in the period between January 2016 and December 2021. The research protocol was approved on 15 December 2021 by the Ethics Committee of the “Victor Babes” University of Medicine and Pharmacy in Timisoara, Romania, with approval number 41, and by the Ethics Committee of the hospitals involved, having approval number 12570.

### 2.2. Inclusion Criteria and Study Variables

A database and patient paper record search were conducted to determine the cases of endocarditis identified based on the ICD-10 classification of diseases [27] and diagnosed following modified Duke criteria for infective endocarditis [28]. A thorough search of electronic databases and paper records was conducted to assess the number of endocarditis infections that hospitalized patients contracted. Electrocardiogram (EKG), imagistic observations, and bacterial identification using traditional cultures and PCR were used to determine the correct diagnosis of infective endocarditis [29,30]. Other more in-depth evaluations were carried out to evaluate the clinical course of the afflicted individuals and to monitor the development of complications. Other patient-specific factors, such as a recent history of dental, maxillofacial, and ENT interventions, as well as catheterization and valvular treatments, were gathered from paper records and database records, being well-known risk factors [31,32,33,34,35]. Patients who lacked consent or had personal data missing key information for the current study were excluded from data collection. In addition, other exclusion criteria were patient age less than 18 years old and bacterial identification showing multiple bacterial infections. The follow-up time of patients identified was the duration of hospitalization. Patients were separated into two groups based on the Gram staining of the bacteria involved, in order to compare the severity of IE, outcomes, and antimicrobial resistance pattern between these two classes of bacteria.

The variables considered relevant to the current study comprised (I) patients’ demographics and background: mean age, gender (men, women), body mass index (underweight, normal weight, overweight), place of origin (urban, rural), occupation (employed, unemployed, retired), living conditions (independent living, institutional care), substance use behavior (smoking, frequent alcohol consumption, drug user), comorbidities (cardiovascular disease, valvulopathies, diabetes mellitus, cerebrovascular disease, digestive and liver disease (chronic hepatitis, cirrhosis, fatty liver disease, gastritis, peptic ulcer, diverticular disease, inflammatory bowel disease), chronic kidney disease, cancer, immunosuppression, bad oral hygiene (defined as a lack of regular teeth brushing, tooth decay with or without gingivitis, and periodontal disease), and other comorbidities); (II) features of the infection (diagnostic delay, empiric treatment delay, presence of vegetations, presence of cardiac abscess), predisposing valvulopathies (aortic, mitral, tricuspid), presence of pacemaker device, etiology of infection (peripheral or central vein catheter, hemodialysis, cardiac surgery, angiography, vascular surgery, gastrointestinal disease, maxillo-facial interventions, dental/ear-nose-throat interventions, unknown source); (III) clinical findings and outcomes (signs and symptoms, Duke criteria, cardiac signs, electrocardiogram abnormalities, rheumatic signs, skin findings, renal involvement, hematological abnormalities, severity of valvular regurgitation, intensive care unit admission, duration of ICU stay, duration between first symptoms and ICU admission, days from symptom onset until death, mortality rate, duration of hospitalization; (IV) microbial detection, treatment, and antimicrobial resistance features (tests performed for infection identification, positive and negative results, proportion of severe treatment complications, treatment regimen type, antibiotics side effects, assessment of multidrug resistance, and distribution of antimicrobial resistance. The classes of antibiotics tested for patients included in the current study comprised the following classes available in the hospitals involved: cephalosporins, penicillins, glycopeptides, aminoglycosides, macrolide, quinolones, tetracycline, carbapenems, and nitroimidazoles. The etiology was established by pathogen isolation when possible and always by the review of an expert cardiologist in correlation with a preceding event that is a known risk factor. Diagnostic delay was considered as the time elapsed from first symptoms until positive blood culture or positive Duke criteria.

### 2.3. Methods of Microbial Detection and Antibiotic Resistance

Microbial identification was performed using the VITEK^®^ 2 system (bioMérieux, Inc, Hazelwood, MO, USA) from blood samples retrieved at admission, followed by blood cultures and microscopic evaluation of Gram-stained blood samples. The typical plate culture is a qualitative and quantitative method that depends on the sample being placed in or on an agar layer that is contained inside a Petri dish. Different types of agar medium were used to cultivate and count bacterial colonies and assess their sensitivity to antibiotics [36]. Antimicrobial resistance was evaluated using disk diffusion and minimum inhibitory concentration techniques, as well as phenotypic and genotypic characterization of bacterial resistance [37].

The disk diffusion procedure was performed based on the diffusion of an antimicrobial agent of a defined concentration from disks, tablets, or strips into a solid culture medium implanted with a pure culture of a particular inoculum [38]. The antibiotics used to test the resistance of Gram-positive bacteria comprised vancomycin, penicillin, ceftriaxone, cefotaxime, ampicillin/amoxycillin, ciprofloxacin, levofloxacin, gentamicin, erythromycin, tetracycline, clindamycin, linezolid, fosfomycin, and imipenem/meropenem. For Gram-negative bacteria, the antibiotics used for testing included meropenem/imipenem, cephalosporins (cefepime, ceftriaxone, ceftazidime), fluoroquinolone (ciprofloxacin, levofloxacin), clarithromycin, amoxycillin, gentamicin, ampicillin/piperacillin, sulbactam/tazobactam, and colistin.

Bacterial identification was performed using a multiplex PCR assay [39]. The PCR multiplex methodology was executed using 1 mL of blood, then centrifuged for 5 min. Protein precipitate is proceeded by DNA precipitation with isopropanol, drying, and resuspension of the DNA pellet. Before elution, the liberated DNA in the QIAEX II Gel Extraction kits (Qiagen, Inc, Hilden, Germany) is attached to a silica gel membrane and a glass fiber filter, and then it is washed. The DNA generated by the lysis buffer is affixed to glass-coated magnetic beads, which the equipment transfers via a series of washing procedures. The DNA is finally eluted, and the beads are disposed of. The only exception was that the final volume of the DNA preparation was always adjusted using the elution or resuspension buffer supplied with each kit [40]. Total DNA was retrieved from the pellet, followed by PCR testing using a standard PCR approach on a Gene Amp PCR System 9700 thermal cycler. Amplification products were purified using the QIAEX II Gel Extraction Kit (manufactured by Qiagen, Shanghai, China) before being sequenced for analysis (BMR Genomics, Padua, Italy).

### 2.4. Statistical Analysis

The statistical analysis was performed with IBM SPSS v.27 (SPSS. Inc., Chicago, IL, USA) [41], while the significance threshold was set for an alpha value of 0.05. The absolute and relative frequencies of categorical variables were computed and compared using the Chi-square and Fisher’s tests. The normality of data was compared using the Kolmogorov–Smirnov test. Parametric continuous variables that followed a normal distribution were compared by mean and standard deviation with the Student’s *t*-test. The Mann–Whitney U-test test was used to compare mean rank differences among nonparametric variables. A Kaplan–Meier analysis was performed to determine the probability of mortality based on infection with Gram-negative or Gram-positive bacteria and by the pattern of antibiotic resistance. A Cox proportional hazard model was used to calculate the hazard ratio for mortality.

## 3. Results

### 3.1. Patients’ Demographics and Background

During the study period, the database search identified 29 patients diagnosed with infective endocarditis mono-infection with a Gram-negative bacterium and 142 cases of mono-infection endocarditis with a Gram-positive bacteria. The background characteristics and patients’ demographics presented in Table 1 identified no significant difference between the mean age of participants and patients’ gender. Patients in the Gram-negative group had a mean age of 62.6 years, compared to 60.9 years in the Gram-positive group. The participant’s body mass index was significantly different between groups. In the Gram-negative group, 17.2% of patients were underweight, compared with only 8.5% in the Gram-positive group. However, this difference might be correlated with the other characteristics of the patients, where 11 (37.9%) of those with Gram-negative IE were living under institutional care, compared with 16 (11.3%) in the Gram-positive group (*p*-value < 0.001).

Other characteristics included the patients’ substance use behavior, observing that a significantly higher proportion of patients in the Gram-positive group were smokers (36.6% vs. 17.2%, *p*-value = 0.043). Patients’ comorbidities with significant differences between groups were cerebrovascular diseases, chronic kidney disease, and bad oral hygiene, all of which are more common among those with Gram-negative IE (*p*-value < 0.05).

### 3.2. Endocarditis Features

It was determined that there were no significant differences in diagnostic delays, the onset of empiric treatment, the presence of vegetations, and the presence of cardiac abscesses between the two infection groups. Vegetations were documented in patients’ records in 65.5% of Gram-negative infections and 73.2% of Gram-positive infections (*p*-value = 0.399). However, the predisposing valvulopathies were significantly different in group proportions, where 4 (80.0%) IE patients in the Gram-negative group had aortic valve disease, compared with 22 (64.7%) patients in the Gram-positive group with mitral valve disease (*p*-value = 0.034). However, the difference might be attributed to the very small sample size in the Gram-negative patient group. Another important study variable was the etiology of infections, observing that a significantly higher number of Gram-negative infections were associated with peripheral and central venous catheter use (31.0% vs. 12.7%, *p*-value = 0.013). Although the other sources of infection documented in patients’ records did not differ significantly, it was observed that many patients with IE with a Gram-negative pathogen were diagnosed after undergoing a maxillo-facial intervention or dental and ENT procedures (17.2% and 15.5%, respectively). The source of infection was unknown in a total of 23 patients, as presented in Table 2.

Table 3 described the clinical findings and outcomes of patients with Gram-negative and Gram-positive IE. It was observed that significantly fewer patients with Gram-negative endocarditis presented with fever (79.3% vs. 93.0%, *p*-value = 0.021) and constitutional symptoms of IE (62.1% vs. 80.3%, *p*-value = 0.033). However, the neurological involvement was significantly more prevalent among those with Gram-negative IE (44.8% vs. 26.1%, *p*-value = 0.042), although we cannot rule out the confounding factor of a significantly higher prevalence of cerebrovascular comorbidities among the same patients. Cardiac signs of new onset murmur did not differ significantly, and there was no difference in the EKG abnormalities. However, valvular regurgitation was more severe among those with Gram-negative IE, in correlation with the presence of heart failure signs (48.3% vs. 31.7%, *p*-value = 0.044). Following the regurgitation severity, the ICU admission, ICU hospitalization, and the mortality rate were significantly higher among patients with Gram-negative IE.

### 3.3. Microbial Identification and Antibacterial Management

Most infections were confirmed by a conventional bacterial culture (three cultures for each patient), while those with negative results were tested by PCR, depending on availability. There were no differences in methods of bacterial identification and false-negative test results. Similarly, there were no significant differences between the two study groups in the proportion of antibiotic side effects and severe treatment complications. Resistance to at least one antibiotic was identified in 24 (82.7%) patients with IE from the Gram-negative group, and 101 (71.1%) in the Gram-positive group. The distribution of antimicrobial resistance by Gram-negative and Gram-positive infections, presented in Table 4, also did not identify any significant differences. There were 11 (37.9%) patients with Gram-negative IE who showed resistance to three or more antimicrobials, and 40 (28.2%) with Gram-positive infections.

**Figure 1:**
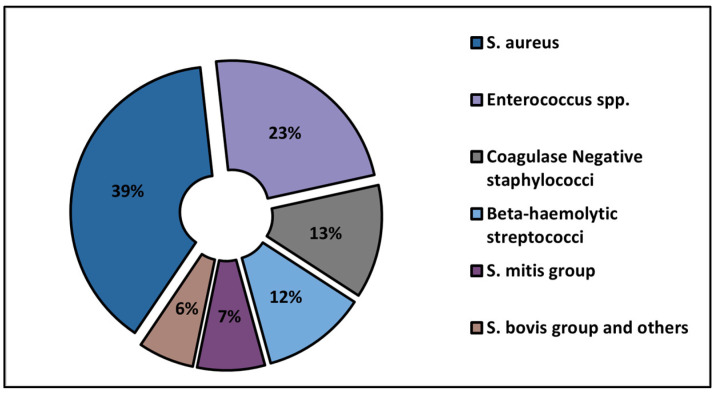
The proportion of Gram-positive bacteria identified.

**Figure 2:**
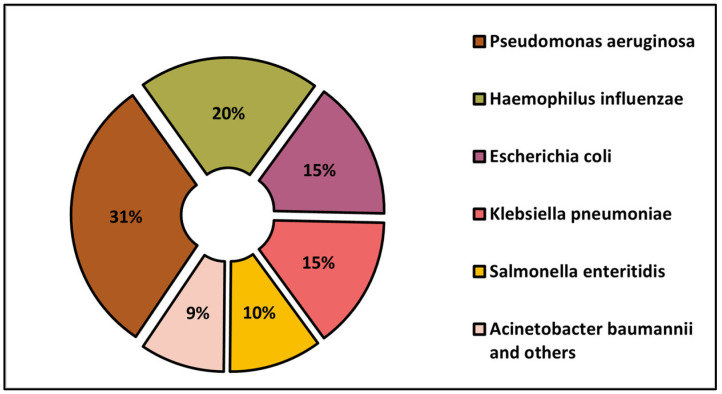
The proportion of Gram-negative bacteria identified.

The proportion of Gram-positive and Gram-negative infections by the identified pathogen is presented in Figure 1 and Figure 2. The most commonly involved Gram-positive pathogen for IE was *S. aureus* (39% of all cases), followed by *Enterococcus* spp. in 23% of patients, and coagulase-negative staphylococci (13%). Among the Gram-negative IE, *pseudomonas aeruginosa* was the most commonly involved (31%), followed by *Haemophilus influenzae* in 20% of patients and *E. coli* in 15%.

### 3.4. Risk Analysis

**Figure 3:**
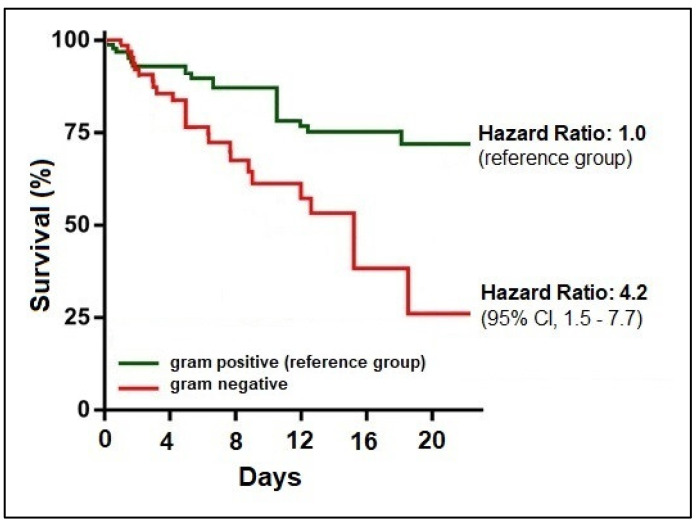
Kaplan–Meier probability curve for mortality by type of infection.

**Figure 4:**
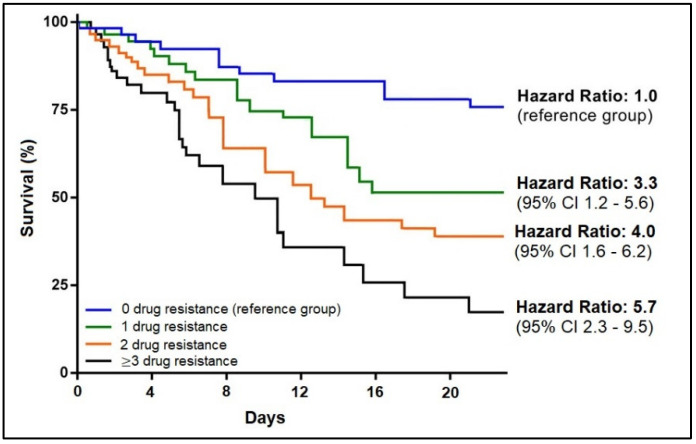
Kaplan–Meier probability curve for mortality by number of antimicrobial resistance.

A Kaplan–Meier mortality curve is shown in Figure 3 and Figure 4 and is stratified by the Gram staining of the bacteria involved and by the antimicrobial resistance pattern. The hazard ratio for mortality was 4.2 times higher in patients with infective endocarditis with a Gram-negative pathogen compared to the reference group of Gram-positive infections (95% CI = 1.5–7.7). When comparing the survivability by antimicrobial resistance pattern, it was observed that IE patients with the involving pathogen resistant to one specific antibiotic had a 3.3-fold higher likelihood of death. For resistance to two antimicrobials, the hazard ratio was 4.0 (95% CI = 1.6–6.2), and for resistance to three or more antibiotics, the mortality risk was 5.7 times higher than the reference group with no drug resistance to specific antibiotics (95% CI = 2.3–9.5).

**Figure 5:**
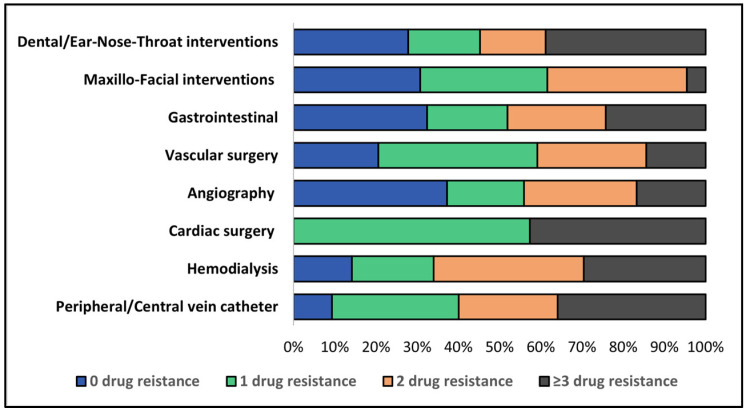
Stacked bar representation of antimicrobial resistance by the source of infection.

A matrix with the pattern of antimicrobial resistance by etiology of infection is described in Table 5. It was observed that Gram-negative pathogens involved in IE were 37.9% resistant to three or more specific antimicrobials, while 20.7% were sensitive to all specific antibiotics. In the Gram-positive group of infections, where *S. aureus* was the most prevalent pathogen, the multi-resistant strains to three or more specific antibiotics were present in 11.2% of patients, while 27.4% of all pathogens showed similar resistance patterns. By source of infection, it was observed that the most frequent etiology of IE with pathogens resistant to three or more specific antimicrobials were peripheral catheters, followed by cardiac surgery and dental and ENT interventions (Figure 5).

## 4. Discussion

### 4.1. Important Findings

The current study provides an important insight into the features of pathogens involved in infective endocarditis, including the antimicrobial resistance pattern of these pathogens and the correlation between the etiology of the infection and antibiotic sensitivity. The study’s main takeaways are that patients from this populational group admitted with Gram-negative infective endocarditis are at a significantly higher risk of complications, ICU admissions, and mortality than patients with Gram-positive infections. Nevertheless, the antimicrobial resistance pattern did not differ significantly between these two categories. However, the results should be weighted for the possible biasing factors, since those with Gram-negative infections had more predisposing comorbidities, were more frequently living in institutional care, and were more frequently underweight. Still, many pathogens involved in IE seem to have a high resistance to specific antimicrobials, regardless of their characteristics and Gram staining. One hypothesis that could explain the high antibiotic resistance in this population of patients with IE is that there can be a reservoir of multidrug-resistant germs in rural areas with industrial animal fattening owing to the overuse of antibiotics to keep the animals alive during growth [42,43]. Considering approximately 60% of patients in our study were residing in urban areas, where they might work in the nearby farms and consume the meat of these animals, they may have been colonized with these multi-resistant germs.

Even when treated with effective antibiotic regimens, IE is still linked with substantial morbidity and death. Treatment is determined by the causing infection, its antibiotic susceptibility profile, local and systemic consequences, and the presence of prosthetic materials or devices. The standard treatment consists of four to six weeks of intravenous antibacterial therapy. However, in other circumstances, intravenous antibiotic treatment may be difficult owing to expense, the difficulty of IV access, the severity of side effects of the prescription, or overuse concerns [44]. Recently, one study demonstrated positive results for partial oral antibiotic treatment regimens administered to patients with clinically stable and complication-free Gram-positive IE involving staphylococcal, streptococcal, and enterococcal IE. Before suggesting the regular use of oral antibiotics in treating individuals with IE, more research should be conducted to better characterize eligible patients and the usage regimens available in countries that have adopted this practice [45].

In the current study, the severity and proportion of complications after antibiotic treatment did not differ significantly between the groups of patients with Gram-positive and Gram-negative IE, although the percentage was high overall. In these cases, the severity of infection and mortality risks usually overcome the possible side effects of aggressive antibiotic treatment. Recent studies indicate, however, that IV antibiotics may not be required for the whole therapy period depending on the clinical conditions. Extended courses of intravenous antibiotics are linked with many hazards and high costs, including procedural problems from intravenous lines, central-line-related infections, and thrombosis [6].

One study demonstrated non-inferior results for patients moved to oral antibiotics when a strong clinical response had been established while on IV medication. However, the retrospective nature of the study and the wide range of days preceding the transition made it difficult to apply the findings. Another prospective randomized trial of patients with Gram-positive left-sided IE offers greater evidence that in some individuals, properly selected oral antimicrobial regimens generate non-inferior results in comparison to all-IV treatment [46,47]. However, the results were not studied on IE patients with Gram-negative infections, as described in our study, likely due to the rarity of such cases.

Regarding the pathogens most frequently found on bacterial cultures in IE reported in a multitude of studies, *S. aureus* remains the most prevalent pathogen involved in Gram-positive bacterial endocarditis. The correlation between the prevalence of *S. aureus* infection and deteriorating clinical outcomes in infective endocarditis is becoming more evident [48,49]. Similarly, in our research, during the six-year period of study, *S. aureus* was the pathogen most commonly found among Gram-positive pathogens. *S. aureus* is a pathogen with a reputation for aggression; it is often linked with severe clinical manifestations. Although our study did not report the complications of endocarditis, in particular, other authors have reported cerebral embolism in 30% of patients with *S. aureus* infective endocarditis [50]. Although embolism was more frequently associated with this bacteria, there was no significant difference in the vegetation size measured between other bacterial groups.

Other wider studies reported different results, which shows that one study alone cannot be representative of a global population of IE patients, and studies should be included in a meta-analysis to draw proper conclusions. For example, in a Chinese study, IE patients had an average age of 46 years old, which was much lower than the average age of patients in our study and those from more developed regions, which ranges between an average of 55 and 70 years old [51,52]. The decline in the prevalence of congenital heart disease and rheumatic heart disease, as well as the rise in the prevalence of degenerative valve disease, are the factors that have contributed to the upward trend of patients in Europe who have IE [53].

The most important diagnostic factor for infectious endocarditis was a blood culture; even though the PCR technique is more accurate and faster, the associated availability and costs make it an unsustainable diagnostic method, especially in poorer regions. According to a recent study in southeast Asia, the *Viridans* group streptococci were the most frequent pathogen involved in IE [54]. In developed regions of the world, *S. aureus* was shown to be the most prevalent causal pathogen, as identified in our study [49]. Additionally, some other studies found that the particular bacterium that was responsible for the infection changed depending on the setting in which it was acquired, with Staphylococcus aureus being the most prevalent strain among infections that were contracted from medical facilities. In our research, the low prevalence of staphylococcus infections might be attributed to the following causes. These risk factors included drug addiction, interaction with medical professionals, and invasive procedures, among others [55]. Second, those who had poor tooth health were more likely to have *Viridans* group Streptococci as their pathogen of choice, being a reflection of the poor oral hygiene that exists among certain population groups.

### 4.2. Study Limitations and Future Perspectives

One limitation of the current study is the retrospective design, which comes with a high risk of encountering biasing factors such as data collection bias, sampling bias, and misclassification bias. In addition, the sample size might need to be increased to draw conclusions applicable at a populational level, since the group of Gram-negative IE patients comprised only 29 patients. Therefore, the statistical power of the study is reduced. Further epidemiological studies that comprise a wider range of years are needed to evaluate the evolution of pathogens involved in infective endocarditis and study the resistance pattern of these bacteria. Another research perspective is the study of sources of infection in correlation with antibiotic resistance. As the population structure is in constant evolution, and more elderly patients are residing under institutional care, it seems that the correlation of being underweight and having poor dentition and oral hygiene predispose patients to severe infections with Gram-negative pathogens, as found in the current study. Therefore, further research is needed in this direction. Additionally, it is important to compare the current findings with other populational studies on IE to be able to identify all risk factors and provide careful prophylaxis.

## 5. Conclusions

Infective endocarditis caused by Gram-negative bacteria determined a higher mortality rate than Gram-positive IE in the studied population. However, these findings should be adjusted for the small sample size of gram-negative IE cases as well as potential biasing factors, since patients with Gram-negative infections had a higher proportion of comorbidities and predisposing factors such as residing in institutional care and being underweight. Although the severity of IE was considerably higher where Gram-negative pathogens were involved, having worse outcomes, more ICU admissions, and a higher fatality rate, the resistance pattern between Gram-negative and Gram-positive bacteria was not significantly different. Another important conclusion is that many IE-associated bacteria seem to be highly resistant, regardless of their category. As the population structure is constantly changing and an increasing number of elderly patients are returning to institutional care, it seems that being underweight and having poor dentition and bad oral hygiene predispose them to Gram-negative IE, associated with a worse prognosis. To analyze the development of pathogens implicated in infectious endocarditis and examine the resistance pattern of these bacteria, further epidemiological studies over a longer time span are required. This is another alarming sign, showing the growing antibiotic resistance that predicts increased mortality rates if alternatives are not discovered in the near future.

## Figures and Tables

**Table 1:** Patients’ demographics and background.

Variables	Gram-Negative (*n*= 29)	Gram-Positive (*n* = 142)	Significance
Age (mean ± SD)	62.6 ± 8.1	60.9 ± 7.8	0.289 *
Sex (Men)	17 (58.6%)	86 (60.6%)	0.845
BMI			0.042
Underweight (<18.5 kg/m^2^)	5 (17.2%)	12 (8.5%)	
Normal weight (18.5–25.0 kg/m^2^)	16 (55.2%)	56 (39.4%)	
Overweight (>25.0 kg/m^2^)	8 (27.6%)	74 (52.1%)	
Place of origin (urban)	17 (58.6%)	82 (57.7%)	0.930
Occupation			0.342
Employed	1 (51.3%)	12 (8.5%)	
Unemployed	10 (42.3%)	33 (23.2%)	
Retired	18 (42.3%)	97 (68.3%)	
Living conditions			<0.001
Independent living	18 (62.1%)	126 (88.7%)	
Institutional care	11 (37.9%)	16 (11.3%)	
Substance use behavior			
Smoking	5 (17.2%)	52 (36.6%)	0.043
Frequent alcohol consumption	2 (6.9%)	29 (20.4%)	0.084
Injection drug use	0 (0.0%)	2 (1.4%)	0.520
Non-injection drug use	0 (0.0%)	11 (7.7%)	0.121
Comorbidities			
Cardiovascular disease (non-valvular)	13 (44.8%)	59 (41.5%)	0.744
Valvulopathies	5 (17.2%)	34 (23.9%)	0.433
Diabetes mellitus	5 (17.2%)	47 (33.1%)	0.091
Cerebrovascular disease	8 (27.6%)	17 (12.0%)	0.030
Digestive and liver	4 (13.8%)	30 (21.1%)	0.367
Chronic kidney disease	12 (41.4%)	32 (22.5%)	0.034
Bad oral hygiene	17 (58.6%)	55 (38.7%)	0.048
Cancer	1 (3.4%)	8 (5.6%)	0.631
Immunosuppression	3 (10.3%)	11 (7.7%)	0.641
Others	2 (6.9%)	6 (4.2%)	0.534

Data reported as *n* (%) and calculated using the Chi-square test and Fisher’s exact test unless specified differently; * Computed with Student’s *t*-test; BMI—Body Mass Index; SD—Standard deviation.

**Table 2:** Characteristics of the infection between patients with Gram-negative and Gram-positive infections.

Variables	Gram-Negative (*n* = 29)	Gram-Positive (*n* = 142)	Significance
Diagnostic delay, days (median, IQR)	2.5 (2.0)	3.5 (2.5)	0.103
Empiric treatment delay, days (median, IQR)	1.5 (2.5)	2.0 (2.0)	0.322
Presence of vegetations	19 (65.5%)	104 (73.2%)	0.399
Presence of cardiac abscess	3 (10.3%)	31 (21.8%)	0.157
Predisposing valvulopathy (*n* = 39)	(*n* = 5)	(*n* = 34)	0.034
Aortic	4 (80.0%)	8 (23.5%)	
Mitral	1 (20.0%)	22 (64.7%)	
Tricuspid	0 (0.0%)	4 (11.8%)	
Presence of a pacemaker device	1 (3.4%)	26 (18.3%)	0.045
Etiology			
Peripheral/Central vein catheter	9 (31.0%)	18 (12.7%)	0.013
Hemodialysis	1 (3.4%)	11 (7.7%)	0.408
Cardiac surgery	0 (0.0%)	4 (2.8%)	0.360
Angiography	2 (6.9%)	31 (21.8%)	0.063
Vascular surgery	2 (6.9%)	10 (7.0%)	0.977
Gastrointestinal	1 (3.4%)	18 (12.7%)	0.149
Maxillo-Facial interventions	5 (17.2%)	14 (9.9%)	0.249
Dental/Ear-Nose-Throat interventions	6 (15.5%)	18 (12.7%)	0.257
Unknown	3 (10.3%)	20 (14.1%)	0.590

Data reported as *n* (%) and calculated using the Chi-square and Fisher’s exact test unless specified differently; IQR—Interquartile range.

**Table 3:** Clinical findings and outcomes of patients with Gram-negative and Gram-positive IE.

Variables	Gram-Negative (*n* = 29)	Gram-Positive (*n* = 142)	Significance
Signs and symptoms			
Fever	23 (79.3%)	132 (93.0%)	0.021
Constitutional symptoms	18 (62.1%)	114 (80.3%)	0.033
Neurological involvement	13 (44.8%)	37 (26.1%)	0.042
Duke criteria			<0.001
Two major criteria	21 (72.4%)	46 (32.4%)	
One major + three minor criteria	5 (17.2%)	74 (52.1%)	
Five minor criteria	3 (10.3%)	22 (15.5%)	
Cardiac signs			
Heart failure	14 (48.3%)	45 (31.7%)	0.044
Murmur	16 (55.2%)	68 (47.9%)	0.474
EKG abnormalities	18 (62.1%)	75 (52.8%)	0.138
Embolic manifestations	8 (27.6%)	31 (21.8%)	0.501
Rheumatic signs	9 (31.0%)	74 (52.1%)	0.038
Skin findings	3 (10.3%)	29 (20.4%)	0.204
Renal involvement	19 (65.5%)	94 (59.2%)	0.523
Hematological abnormalities	13 (44.8%)	45 (31.7%)	0.173
The severity of valvular regurgitation			0.036
Mild	6 (20.7%)	54 (38.0%)	
Moderate	14 (48.3%)	69 (48.6%)	
Severe	9 (31.0%)	19 (13.4%)	
Outcomes			
ICU admission	19 (65.5%)	63 (44.4%)	0.037
Days in the ICU (mean ± SD)	5.6 ± 2.4	7.9 ± 3.1	<0.001
Days between symptom onset until death (mean ± SD)	6.2 ± 3.0	7.4 ± 3.9	<0.001
Days between symptom onset until ICU admission (median, IQR)	5.8 ± 4.1	3.0 ± 5.3	<0.001
Mortality	17 (58.6%)	49 (34.5%)	0.015
Days until discharge (mean ± SD)	19.6 ± 5.3	14.4 ± 6.0	<0.001

Data reported as *n* (%) and calculated using the Chi-square test and Fisher’s exact test unless specified differently; ICU—Intensive Care Unit; SD—Standard Deviation; EKG—Electrocardiogram.

**Table 4:** Microbial detection, treatment, and antimicrobial resistance features.

Variables *	Gram-Negative (*n* = 29)	Gram-Positive (*n* = 142)	Significance
Tests performed for infection identification			
Conventional culture	24 (82.8%)	130 (91.5%)	0.149
PCR	5 (17.2%)	12 (8.5%)	0.149
Culture and PCR	7 (24.1%)	36 (25.4%)	0.890
Testing			0.435
Positive samples	17 (58.6%)	94 (66.2.%)	
False negative result	12 (41.4%)	48 (33.8%)	
Antibiotic therapy			0.619
Intravenous	25 (86.2%)	111 (78.2%)	
Oral	3 (10.3%)	12 (16.2%)	
Combination	1 (3.4%)	36 (5.6%)	
Severe treatment complications	7 (24.1%)	21 (14.8%)	0.215
Treatment regimen type			0.361
Monotherapy	11 (37.9%)	67 (47.2%)	
Combined	18 (62.1%)	75 (52.8%)	
Change in antibiotics during hospitalization	8 (27.6%)	30 (21.1%)	0.445
Antibiotics side effects			
Acute immune reactions	2 (6.9%)	7 (4.9%)	0.665
Delayed reactions	1 (3.4%)	6 (4.2%)	0.847
Nephrotoxicity	9 (31.0%)	29 (20.4%)	0.210
Neurotoxicity	4 (13.8%)	14 (9.9%)	0.529
Liver injury	5 (17.2%)	11 (7.7%)	0.109
Digestive side effects	5 (17.2%)	33 (23.2%)	0.478
Falls and delirium	5 (17.2%)	11 (7.7%)	0.109
Multidrug resistance			0.368
Yes	25 (86.2%)	130 (91.5%)	
No	4 (13.8%)	12 (8.5%)	
Distribution of antimicrobial resistance	(*n* = 29)	(*n* = 142)	0.549
0 drug resistance (*n* = 46)	5 (17.2%)	41 (28.9%)	
1 drug resistance (*n* = 33)	5 (17.2%)	28 (19.7%)	
2 drug resistance (*n* = 41)	8 (27.6%)	33 (23.2%)	
≥3 drug resistance (*n* = 51)	11 (37.9%)	40 (28.2%)	

* Data reported as *n* (%) and calculated using the Chi-square test and Fisher’s exact test unless specified differently; PCR—Polymerase Chain Reaction test.

**Table 5:** Antimicrobial resistance pattern by etiology of infection.

Variables	0 Drug Resistance (*n* = 46)	1 Drug Resistance (*n* = 33)	2 Drug Resistance (*n* = 41)	≥3 Drug Resistance (*n* = 51)	Significance
Gram-negative	(*n* = 6)	(*n* = 2)	(*n* = 10)	(*n* = 11)	0.506 *
*Pseudomonas aeruginosa* (*n* = 9)	1 (16.7%)	1 (34.3%)	3 (30.0%)	4 (36.4%)	
*Haemophilus influenzae* (*n* = 6)	2 (33.3%)	0 (34.3%)	2 (20.0%)	2 (18.2%)	
*Escherichia coli* (*n* = 4)	1 (16.7%)	1 (34.3%)	1 (10.0%)	1 (9.1%)	
*Klebsiella pneumoniae* (*n* = 4)	2 (33.3%)	0 (0.0%)	2 (20.0%)	0 (0.0%)	
*Salmonella enteritidis* (*n* = 3)	0 (0.0%)	0 (0.0%)	2 (20.0%)	1 (9.1%)	
*Acinetobacter baumannii* and others (*n* = 3)	0 (0.0%)	0 (0.0%)	0 (0.0%)	3 (27.3%)	
Gram-positive	(*n* = 40)	(*n* = 31)	(*n* = 31)	(*n* = 40)	0.794 *
*S. aureus* (*n* = 55)	11 (32.4%)	14 (42.4%)	15 (40.5%)	15 (34.9%)	
*Enterococcus* spp. (*n* = 32)	6 (25.0%)	8 (24.2%)	8 (21.6%)	10 (23.3%)	
Coagulase-negative staphylococci (*n* = 18)	4 (8.3%)	3 (9.1%)	6 (16.2%)	5 (11.6%)	
Beta-hemolytic streptococci (*n* = 17)	5 (5.6%)	2 (6.1%)	4 (10.8%)	6 (14.0%)	
*S. mitis* group (*n* = 10)	2 (8.3%)	3 (9.1%)	1 (2.7%)	4 (9.3%)	
*S. bovis* group and others (*n* = 10)	3 (5.6%)	1 (2.9%)	3 (9.1%)	3 (7.0%)	
Etiology	(*n* = 46)	(*n* = 33)	(*n* = 41)	(*n* = 51)	0.328 *
Peripheral/Central vein catheter (*n* = 27)	3 (6.7%)	7 (21.9%)	6 (17.1%)	11 (25.6%)	
Hemodialysis (*n* = 12)	2 (4.4%)	2 (6.3%)	4 (11.4%)	4 (9.3%)	
Cardiac surgery (*n* = 4)	0 (0.0%)	2 (6.3%)	0 (0.0%)	2 (4.7%)	
Angiography (*n* = 33)	14 (31.1%)	5 (15.6%)	8 (22.9%)	6 (14.0%)	
Vascular surgery (*n* = 12)	3 (6.7%)	4 (12.5%)	3 (8.6%)	2 (4.7%)	
Gastrointestinal (*n* = 19)	7 (15.6%)	3 (9.4%)	4 (11.4%)	5 (11.6%)	
Maxillo-Facial interventions (*n* = 19)	7 (15.6%)	5 (15.6%)	6 (17.1%)	1 (2.3%)	
Dental/Ear-Nose-Throat interventions (*n* = 24)	9 (20.0%)	4 (12.5%)	4 (11.4%)	8 (27.9%)	
Unknown (*n* = 23)	–	–	–	–	

* Proportions compared with the Chi-square test and Fisher’s exact test if the expected frequency assumption was not met.

## Data Availability

Data available on request.
